# The call for a strategic framework to improve cancer literacy in Europe

**DOI:** 10.1186/s13690-020-00441-y

**Published:** 2020-06-23

**Authors:** Kristine Sørensen, Lydia E. Makaroff, Laurie Myers, Paul Robinson, Geoffrey J. Henning, Cathryn E. Gunther, Alexander E. Roediger

**Affiliations:** 1 Global Health Literacy Academy, Risskov, Denmark; 2 World Bladder Cancer Patient Coalition, Brussels, Belgium; 3 Fight Bladder Cancer, Chinnor, UK; 4 Merck & Co., Inc., Kenilworth, NJ USA; 5 MSD Ltd., London, UK; 6 Digestive Cancers Europe, Salisbury, UK; 7 MSD International GmbH, Kriens, Switzerland

**Keywords:** Cancer, Cancer literacy, Health literacy, Shared decision-making, Patient engagement, Empowerment, Health outcomes

## Abstract

**Background:**

Health literacy is increasingly being recognized as a widespread public health challenge in Europe. This commentary explores the importance of health literacy amongst cancer patients (ie, cancer literacy) and examines how cancer literacy can be improved through the practical application of health literacy principles within the context of providing timely, patient-centered, value-based care in Europe.

**Main body:**

Despite implementation of evidence-based cancer prevention programs and increased cancer survival rates, low cancer literacy may impact the personal capacity to manage risks and adversely impact behavior and outcomes. Cancer literacy poses a unique set of challenges compared to other types of health literacy, as patient decisions regarding screening, treatment, and side effect management are often complex, and timely decision-making is more critical. Accordingly, European health policies increasingly recognize the importance of health literacy. The European Patients Forum, European Cancer Patient Coalition, and the Association of European Cancer Leagues supported a joint statement, “Europe Let’s Do More for Health,” which emphasizes the need to empower citizens and patients by addressing health literacy, self-management, and shared decision-making. Implementation of comprehensive programs and strategies will be important to improve health literacy. Cancer literacy can be improved through application of health literacy principles in the communication and cooperation with professionals, patients and caregivers for providing timely, patient-centered, value-based care. Recommendations are made for further action to improve cancer literacy in Europe through coordinated efforts among providers, organizations, patients, and research. A policy paper developed by the European Joint Action on Cancer Control provides practical recommendations that Member States can take to reduce social inequalities in cancer care and defines focus areas that are closely connected with the need to improve cancer literacy.

**Conclusion:**

Improved personal cancer literacy combined with health literate organizations and systems can potentially improve the quality of care and health outcomes among patients with cancer. National Cancer Control Plans and Europe’s Beating Cancer Plan can strengthen cancer literacy.

## Background

Disparities related to health literacy are a public health challenge that is increasingly important in Europe today [1]. Health literacy is closely linked to general literacy and entails the knowledge, motivation, and competencies to access, appraise, understand, and apply information for making decisions concerning healthcare, disease prevention and health promotion, and to maintain and improve quality of life during the life course with support from relevant organizational stakeholders [2].

Almost one third or more are thought to have limited health literacy according to European research [1]. With lower levels of health literacy people are at risk of worse health. Subgroups who are particularly vulnerable to limited health literacy include older adults and people with low educational attainment or low socioeconomic status/financial deprivation.

Cancer literacy poses a unique set of challenges compared to health literacy related to other diagnoses and diseases. There are many time-critical decisions to be made by both the patient and provider, since early screening, diagnosis, or starting treatment have an impact on chances to survive. Early detection of cancer greatly increases the chances of survival. Cancer treatments can be complex and involve multiple physicians, tests, treatments, and the need to manage potential side effects. The possibility of early detection using genetic testing, and the difficulties associated with understanding both the test terms and test results, make decisions complex. Thus, any action or lack thereof by patients or providers may have important clinical implications.

This commentary explores the significance of cancer literacy, supports the enhancement of cancer literacy among organizations, providers, patients, and caregivers throughout the cancer continuum, and, therefore, calls for the development of a strategic framework on cancer literacy in the European Union.

### Significance of cancer literacy

Adults with low health literacy are less informed about cancer and more likely to have fatalistic views on cancer and prevention [3, 4]. Low health literacy has also been shown to be associated with avoidance of doctor visits and confusion about screenings [3] as well as with significantly greater apprehension about colorectal cancer screening [4], which may potentially lead to avoidance of screening. People with low health literacy are significantly less likely to report having heard of a colonoscopy, mammography, or prostate-specific antigen test or to correctly identify the cancer being screened for with each test [3].

Cancer literacy is also critical to “navigating the healthcare system” to find the right clinician, follow up about results, and to obtain timely access to treatment or clinical trials. Complex and sophisticated cancer treatments may be difficult to understand even for patients with sufficient health literacy. Among 1060 newly diagnosed breast cancer patients in Germany surveyed immediately after surgery and 10 and 40 weeks following, those with limited health literacy had higher unmet information needs and were at a distinct disadvantage in obtaining information over the course of cancer treatment [5]. In addition to influencing screening and treatment decisions, low health literacy can negatively affect the use of end-of-life and palliative care services. Adequate health literacy is important to ensure that interventions and supportive care meet the needs and wishes of the patient. Shared decision-making, with relatives, friends, and loved ones is an important component of end-of-life care plans, and good cancer literacy is necessary during this process. Therefore, despite successful evidence-based cancer prevention programs and increased cancer survival across the general population, people with low cancer literacy appear to have a decreased personal capacity to manage their risks and may be adversely impacted throughout the cancer continuum, which ranges from diagnosis through treatment to recovery, or end-of-life decisions.

## Health literacy and cancer literacy on the European policy agenda

The importance of health literacy is increasingly being recognized in European health policies [6, 7]. The European Commission has released several communications and spearheaded multiple initiatives addressing health literacy [6]. In 2018, the Health Evidence Network (HEN) of the World Health Organization (WHO; Europe) reported on a scoping review highlighting effective health literacy policy-related activity, predominantly in health and education, and proposed areas for future development [7] for policy makers to consider in their respective countries.

The WHO recognizes health literacy as a social determinant of health and improved health literacy as a key goal of public health [8]. A core principle of the WHO European Roadmap for Implementation of Health Literacy Initiatives Through the Life Course includes the importance of patient empowerment, a process through which individuals gain better understanding and control over their own health. Increased health literacy is noted as a key prerequisite to achieving patient empowerment, which can in turn reduce use of health services and healthcare costs.

Existing EU organizations providing information about cancer care have the potential to assist in cancer literacy advocacy. For instance, The European Patients Forum, European Cancer Patient Coalition, and the Association of European Cancer Leagues (among others) supported a joint statement entitled “Europe Let’s Do More for Health #EU4HEALTH” [9]. One key component of this statement is the need to empower citizens and patients. The different dimensions of patient empowerment include health literacy, self-management, and shared decision-making between patients and providers, and these are currently being addressed through stand-alone projects and initiatives. However, the statement identifies the need for a framework to advance the issue of patient and citizen empowerment in a coherent and meaningful manner.

## What can be done to improve cancer literacy in the European Union?

Although several governing bodies and cancer organizations are beginning to recognize the importance of health literacy, and cancer literacy in particular, implementation of programs and strategies for improvement are still needed. Cancer literacy can be improved through application of health literacy principles in communication and cooperation with professionals, patients, and caregivers for providing timely, patient-centered, value-based care. It is necessary that efforts to improve cancer literacy in Europe take place on multiple levels—the organizational level, the provider level, the patient level, and through co-production and research. Recommendations for further actions to improve cancer literacy in Europe are provided in Table 1.

**Table 1:** Options for further action to improve cancer literacy in Europe

Options for further action regarding cancer literacy in Europe	
**1) Create policy frameworks** ● Develop an EU cancer health literacy framework, reflecting on the role of health literacy in cancer care, potential gaps, and recommendations for action, as outlined in this paper. ● Include health literacy as part of the National Cancer Control Plans and Europe’s Beating Cancer Plan. **2) Specific activities to improve health literacy in cancer** ● Improve health literacy education of health professionals as part of clinical and public health capacity building and incorporating health literacy into educational curricula. ● Improve health literacy of patients by providing adequate and timely support as well as active involvement in their disease and self-management procedures. ● Monitor the implementation of cancer literacy in European healthcare settings and gather lessons learned to enhance the health literacy agenda in general, for example, through the establishment of cancer literacy projects. ● Implement a universal precautions approach by assuming that patients may have difficulty comprehending health information and accessing health services and implementing systems to promote better understanding by all patients. ● Evaluate materials according to evidence-based health literacy principles. **3) Further research** ● Study cancer literacy from the perspective of the patient at different stages of cancer care. ● Study cancer literacy in relation to emotional impact and psycho-social distress. ● Strengthen the health system efforts to bridge communication smoothly between primary, secondary, and tertiary levels of healthcare. ● Study the economic advantages of investing in cancer literacy.	

A starting point for the action framework could be the CanCon Joint Action on Cancer Control, a multinational initiative of the European Commission that produced a policy paper recommending concrete actions to reduce social inequalities in cancer care [10]. The policy paper recommended three areas of focus: (1) support capacity-building for cancer control and prevention, (2) promotion of equity in primary and secondary prevention policy, and (3) promotion of equity in access to cancer care and to survivorship and rehabilitation services. These three focus areas are closely connected with the need to improve cancer literacy and can be implemented at both the national and organizational level as part of National Cancer Control Plans. The implementation of these plans should be monitored in healthcare settings and the lessons learned used to enhance the health literacy agenda in general, for example, through the establishment of cancer literacy projects and the inclusion into Europe’s Beating Cancer Plan (https://ec.europa.eu/health/non_communicable_diseases/events/ev_20200204_de). European Union-based cancer organizations can play an important role in addressing this challenge and improving cancer literacy, as they are uniquely positioned to provide education, materials, and tools to providers and clinicians. Health literacy should be considered while providing patients with adequate and timely care and support. Patients should be encouraged to be actively involved in their disease and self-management procedures.

## Conclusion

Low cancer literacy has been shown to hinder patients at every stage of the cancer journey. Empowering the cancer patient is key for a timely and more positive patient experience. Improved cancer literacy combined with the development of health literate organizations and systems can potentially improve patient care along the whole range of the continuum of care, while potentially reducing the cost of unnecessary and inappropriate care. Accordingly, cancer literacy should be reflected in National Cancer Control Plans and Europe’s Beating Cancer Control Plan and be a priority for policy makers when implementing these plans, professional guidelines, and EU policy programs. An EU framework on cancer literacy will support the ultimate goal of better outcomes for all cancer patients.

## Data Availability

Data sharing is not applicable to this article as no datasets were generated or analyzed during the current study.

## Abbreviations

HEN: Health Evidence Network
WHO: World Health Organization
